# Estimating the number and size of phloem sieve plate pores using longitudinal views and geometric reconstruction

**DOI:** 10.1038/srep04929

**Published:** 2014-05-12

**Authors:** Philippe Bussières

**Affiliations:** 1 Institut National de la Recherche Agronomique, UR 1115, Unité de recherche sur les Plantes et Systèmes de culture Horticoles, 84914 Avignon Cedex 9, France

## Abstract

Because it is difficult to obtain transverse views of the plant phloem sieve plate pores, which are short tubes, to estimate their number and diameters, a method based on longitudinal views is proposed. This method uses recent methods to estimate the number and the sizes of approximately circular objects from their images, given by slices perpendicular to the objects. Moreover, because such longitudinal views are obtained from slices that are rather close to the plate centres whereas the pore size may vary with the pore distance from the plate edge, a sieve plate reconstruction model was developed and incorporated in the method to consider this bias. The method was successfully tested with published longitudinal views of phloem of Soybean and an exceptional entire transverse view from the same tissue. The method was also validated with simulated slices in two sieve plates from Cucurbita and Phaseolus. This method will likely be useful to estimate and to model the hydraulic conductivity and the architecture of the plant phloem, and it could have applications for other materials with approximately cylindrical structures.

The pores of the sieve plates (Figs. 1, 2a and 3a) in the sieve tubes of plant phloem are shaped as short tubes. They are strongly suspected to largely control the transfers in the plant phloem because the total transfer section area at their level is small and the cross-sectional area of each pore is small, which results likely in high forces of viscosity. Because of their small size, they can be a barrier to microorganisms^1^. Their number and cross-sectional size can vary considerably according to species, organs and cell age, from less than one to several hundred per square micrometre of sieve plate and from hundredths of micrometres to micrometres^1^,^2^,^3^,^4^,^5^,^6^. Thus, it is important to be able to estimate these numbers and sizes, particularly to confirm that 95% of the hydraulic conductivity in the young tomato fruit pedicel^7^ depends on the pores. More generally, modelling the architecture of the sieve plate could contribute to model fluxes in the tissue.

However, estimating the number and cross-sectional size of the pores is not easy. Under light microscopy, the pores are too small to be measurable and visible, so that even identifying the sieve tubes among the other phloem cells is difficult. In transmission electron microscopy, longitudinal views of the phloem can show the length of the sieve tubes and the pores (Fig. 1), and it is logical to expect that transverse views (Fig. 2a) give the area of the sieve plates and the number and cross-sectional sizes of their pores. However, obtaining transverse views with slices that are generally 0.07 μm thick on a level with a plate is notably improbable because of the small sieve plate thickness (only approximately 0.5–1 μm), whereas the cell length ranges from a few tens to hundreds of microns. Because there are at most only a few sieve tubes in one transverse view in transmission electron microscopy, only one view out of several tens or hundreds can be expected to show one sieve plate. In addition, because the plates are not often exactly perpendicular to the tubes, all plate pores cannot generally be observed in a slice. The literature^5^,^8^ contains only a few transverse views on a level with a sieve plate where all pores are clearly visible. Fig. 2a shows the contours of the cross-sections of the plate and the pores from Soybean petiolar tissue, which were drawn from the main transverse view that was published by Fisher^8^.

Longitudinal views of phloem (Fig. 1) are generally obtained from a tissue slice that does not pass exactly at the levels of the axes of the tube, plate and pores. However, they are near the tube and plate axes because it is easier to identify a cell such as a sieve tube if the pores are visible. Thus, short sections of plates are not observed. Tube or pore images depend on the tube or pore size, the position of the slice from their centre and the slice thickness. Elsewhere, greater tubes or pores have higher chances to be observed in the slice. Therefore, views such as Fig. 1 do not provide much directly useful information to estimate the sizes of the plate and pores and the number of pores.

Because these elements are only approximately cylindrical, it is desirable to use stereological methods^9^ that are applicable regardless of the object shape. However, because these methods appear difficult to adapt to the present problem, whereas the objective is to study the mechanisms using classic flow laws, (e.g., Poiseuille's Law) which are based on perfect shapes such as cylinders, it is preferable to compare the tubes and pores to cylinders with cross-section areas equal to those of the tubes and pores, whose diameter is called equivalent-diameter.

I have recently proposed a method to estimate the numbers and diameter distributions of circles, cylinders and spheres in a matrix from their images, which are obtained by slices that section the matrix perpendicularly to a diameter, when the diameter distribution is perfectly or approximately symmetrical^10^. This method is applicable to opaque or transparent objects relative to matrix. It considers the slice thickness *t* and the lower image detection limit *L*_S_ (when the shortest images cannot be observed). From this method, I developed a method^11^ for planar objects that are only approximately circular with approximately known shapes. This method was successfully tested using simulated longitudinal slices, which were made in the previously reported transverse view from Fisher^8^.

In the here proposed method, these methods are used to estimate the equivalent-diameters of the plate and pores and the number of pores from longitudinal views. However, these methods, which assume representative samples of pores and plates, likely provide biased estimates when such views are obtained from slices that are near the plate centre, if the pore size varies with its distance from the plate edge. Therefore, the statistical relation between the equivalent-diameters of the pores and their distance from the plate edge is estimated, and a plate reconstruction model is developed and introduced in the proposed method to consider this bias. The method was applied to a set of four longitudinal views, which were also published by Fisher^8^ and taken from the same tissue as the previous transverse view from this author. The estimates were notably close to the values observed in Fisher's transverse view. The method was also successfully tested with images that were obtained from simulated slices in two sieve plates with their pores from two other species (Cucurbita maxima and Phaseolus vulgaris), which were published by Mullendore et al.^5^.

## Results

### The proposed method and its mathematical development

**Figure 1:**
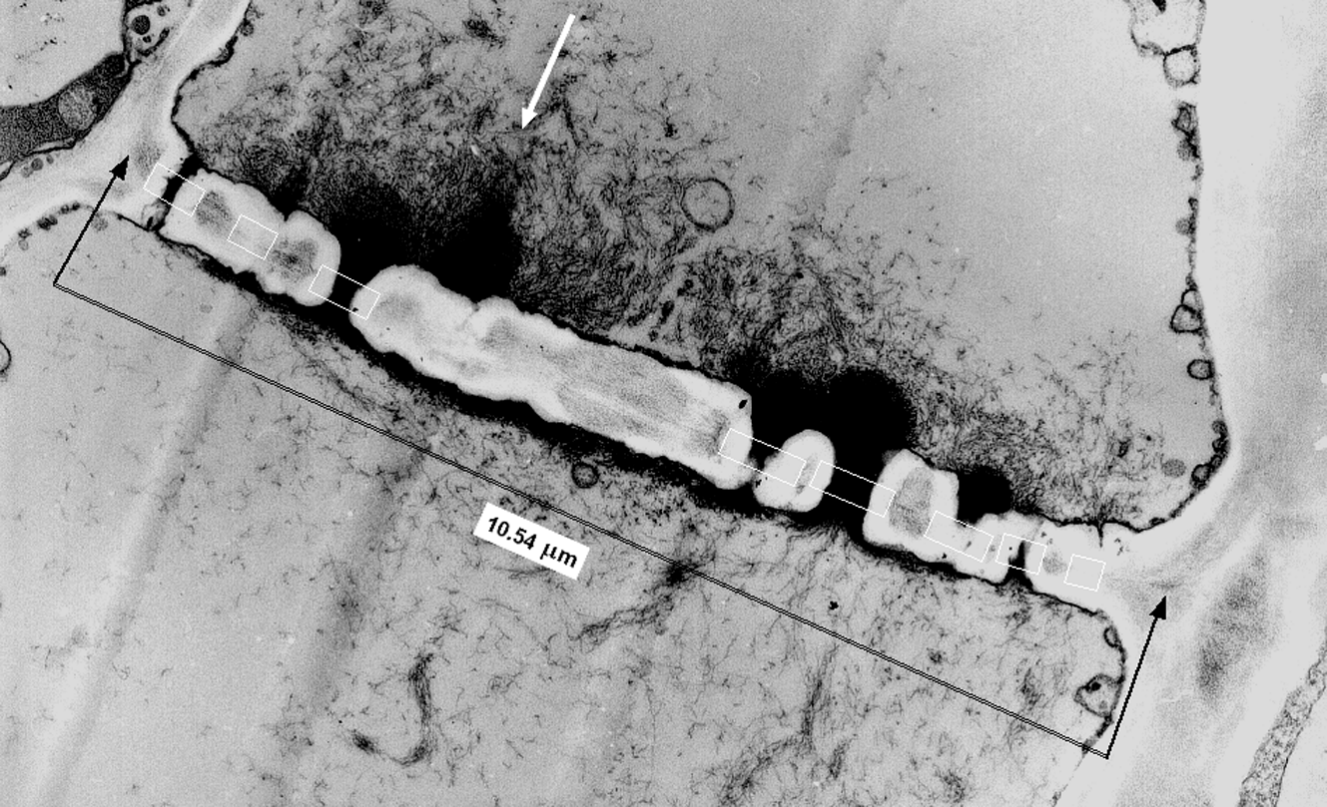
Longitudinal view of a tomato fruit pedicel sieve tube. Sieve tube of pedicel of tomato fruit (weighing 7–15 g) observed under transmission electron microscopy (Philips CM 10) with a magnifying power of 13,000 (fixation by glutaraldehyde and osmium and inclusion in Araldite MCY 212). The distance between the black arrows is 10.54 μm. The mean plate thickness is 0.77 μm. The image lengths of eight pores were measured as the lengths of the sides of the white rectangles along the plate image: from left to right: 0.52, 0.38, 0.61, 0.76, 0.76, 0.58, 0.41 and 0.31 μm. The image light contrast (minimum and maximum values) were modified using Image J to better show the pore edges, but this method does not change the pore image widths. The white arrow shows a site without clear pore image but with a long black zone of likely accumulation proteinaceous material.

**Figure 2:**
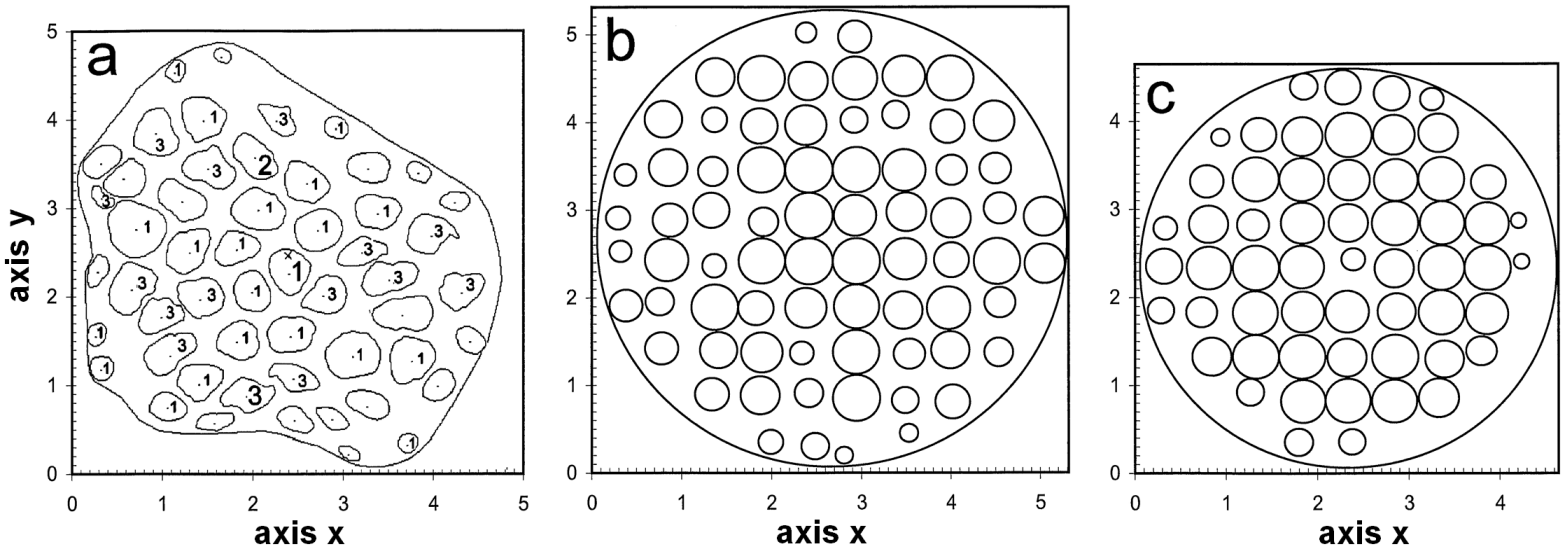
Contours of the plate and pore cross-sections in the transverse view in Fig. 18 from Fisher^8^ (a), and one replication of the transverse plate views which were reconstructed from the four longitudinal views in Fisher^8^ (b) or from Fig. 18 in Fisher^8^ (c). Units of axis: μm. (a) Plate equivalent-diameter: *P* = 4.647 μm. Mean and coefficient of variation of the pore equivalent-diameter: *D*_m_ = 0.405 μm and *cv_D_* = 0.272. Number of pores: 53. Each pore cross-section was approximated by form 1, 2 (without label) or 3. The point in each pore is the centre of the circumscribed circle, which is considered as the pore cross-section centre. (b) For *ν* = 10, *P* = 5.312 μm, *D*_m_ = 0.409 μm and *cv_D_* = 0.226. Number of pores: 72, 11 of which have their diameter reduced to 0.531 μm because it was greater than the plate diameter divided by *ν*. (c) For *ν* = 9, *P* = 4.647 μm, *D*_m_ = 0.424 μm and *cv_D_* = 0.223. Number of pores: 57, 11 of which have their diameter reduced to 0.516 μm because it was greater than the plate diameter divided by *ν*.

**Figure 3:**
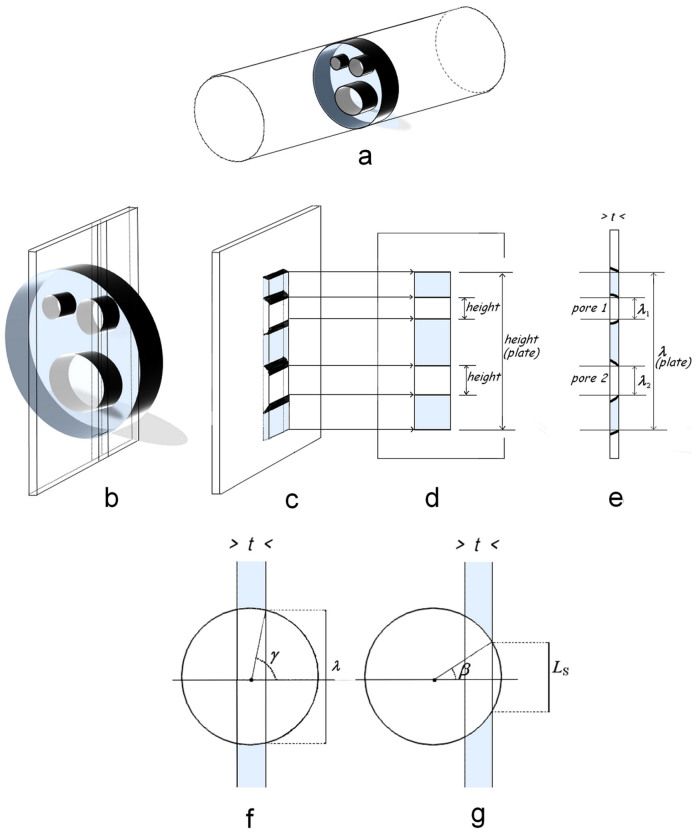
Formation of pore and plate images. A sieve plate, its three pores and the phloem tube are compared to cylinders (a). When a slice longitudinally sections the tube, it is perpendicular to the plate and pore cross-sections (b) and contains pore and plate sections (c). The smallest dimensions of these sections delimit the image size and particularly their height, which are obtained on planes parallel to the slices (d). In the profile view, the image length is equal to the previous height (e). The longest images of a circle with diameter *D* are provided by slices that pass through the circle centre (f), whereas the shorter images are provided by slices at distances shorter than *D*/2 − *t*/2 (f) or much shorter if images smaller than *L*_S_ cannot be observed (g).

The proposed method considers sieve plates with their pores (Figs. 2a and 3a), which are sectioned by slices of thickness *t* perpendicular to the plates (Fig. 3b). When slices (Fig. 3c) are submitted to radiation, they produce 2D images (Figs. 1 and 3d) of the plate and pore sections existing in the slices. These images are comparable to images of transparent objects in an opaque matrix, which are sectioned by slices: their height (Fig. 3d), depends on the smallest object dimension, which delimits the radiation path. In the profile views, these images give lines of equal lengths to the previous height (Fig. 3e). These lines are thus also considered to be images of the pores and plates, and their length *λ* is named the “image length”. The image lengths vary with the plate or pore sizes, the slice thickness *t* and the position of the slice relatively to the plates and pores. For a perfectly cylindrical pore, the images are those of a transparent circle in an opaque matrix sectioned by slices perpendicular the circle. The longest image is obtained when the slice middle passes through the circle centre (Fig. 3f). Images that are shorter than a limit (which is named the lower image detection limit and labelled *L*_S_) cannot be observed (Fig. 3g) because the resolving power of the observation system is too low or, in the case of plate images, only the slices that pass near the plate centre are considered. Because the pores are only approximately cylindrical, the image lengths are only approximately as it was described before.

On views such as Figs. 1 and 3d, the lengths of the plate and pore images and the distances of the pore images to the ends of the plate image can be measured.

The notation of the main variables of image length is provided in Table 1. The capital letter *L* marks the length of an image among the images that are obtained from a single circle or several circles with identical diameter, whereas the small letter *l* marks the length of an image among the images that are obtained from circles with different diameters. Similarly, the capital Greek letter *Λ* and the small greek letter *λ* are for images of pores which are only approximately circular. *L*_m_, *l*_m_ and *λ*_m_ are the means of the lengths *L*, *l* and *λ*, respectively. *D_l_*_m_ is the diameter of the circle of which all images that are obtained from slices spaced by small distances in all possible configurations have for mean length *L*_m_ = *l*_m_. Similarly, *D_λ_*_m_ is the diameter of the circle of which the mean image length, *L*_m_, is equal to *λ*_m_.

#### Estimation of the pore equivalent-diameter distribution and the number of pores per unit plate area, assuming that the pore images are obtained from a sample representative of the plate

First, assume that the cross-sections of all pores have identical form.

Let *λ*_m_ and *cv_λ_* be the mean and the coefficient of variation of the pore image lengths.

From *λ*_m_ and *cv_λ_*, the proposed method estimates, in a first phase, the mean *l*_m_ and the coefficient of variation *cv_l_* of the image lengths that would be obtained if the cross-section of each pore was a perfect circle with identical area as the pore cross-section area, its diameter being called equivalent-diameter of the pore. In a second phase, the method estimates the mean *D*_m_ and the coefficient of variation *cv_D_* of the diameters of these perfect circles from *l*_m_ and *cv_l_* using the previously proposed method to estimate the diameter distribution of circles in a matrix which is sectioned by slices when the diameter distribution is perfectly or approximately symmetrical^10^.

In this later method, *D*_m_ and *cv_D_* are estimated by considering a fictive circle of diameter *D_l_*_m_. This diameter *D_l_*_m_ is calculated so that the mean image length *L*_m_ of this circle over all possible configurations of the slice is equal to the mean length *l*_m_ of the images of the circles provided by the slices. For given values of *t* and *L*_S_, *D_l_*_m_ is calculated from *l*_m_ by successive approximations, based on a number of relations (see Supplementary Note on line) using Supplementary Dataset 1 on line. For these calculations, the smallest and the largest measured image lengths, *L*_min_ and *L*_max_, are assumed to be the central values of the smallest and the largest image length classes. Therefore, *L_S_* is approximated by *L*_min_ − (*L*_max_ − *L*_min_)/(*m* − 1)/2, where *m* is the number of images.

Here, it is assumed that, if the pore cross-sections are sufficiently approximately circular, the cross-section area of a fictive pore that generates the mean image length *λ*_m_ is approximately equal to the area of a fictive circle of diameter *D_l_*_m_, that generates the mean image length *L*_m_ equal to the mean length *l*_m_ of the images of all circles with identical areas to the pore cross-sections.

**Figure 4:**
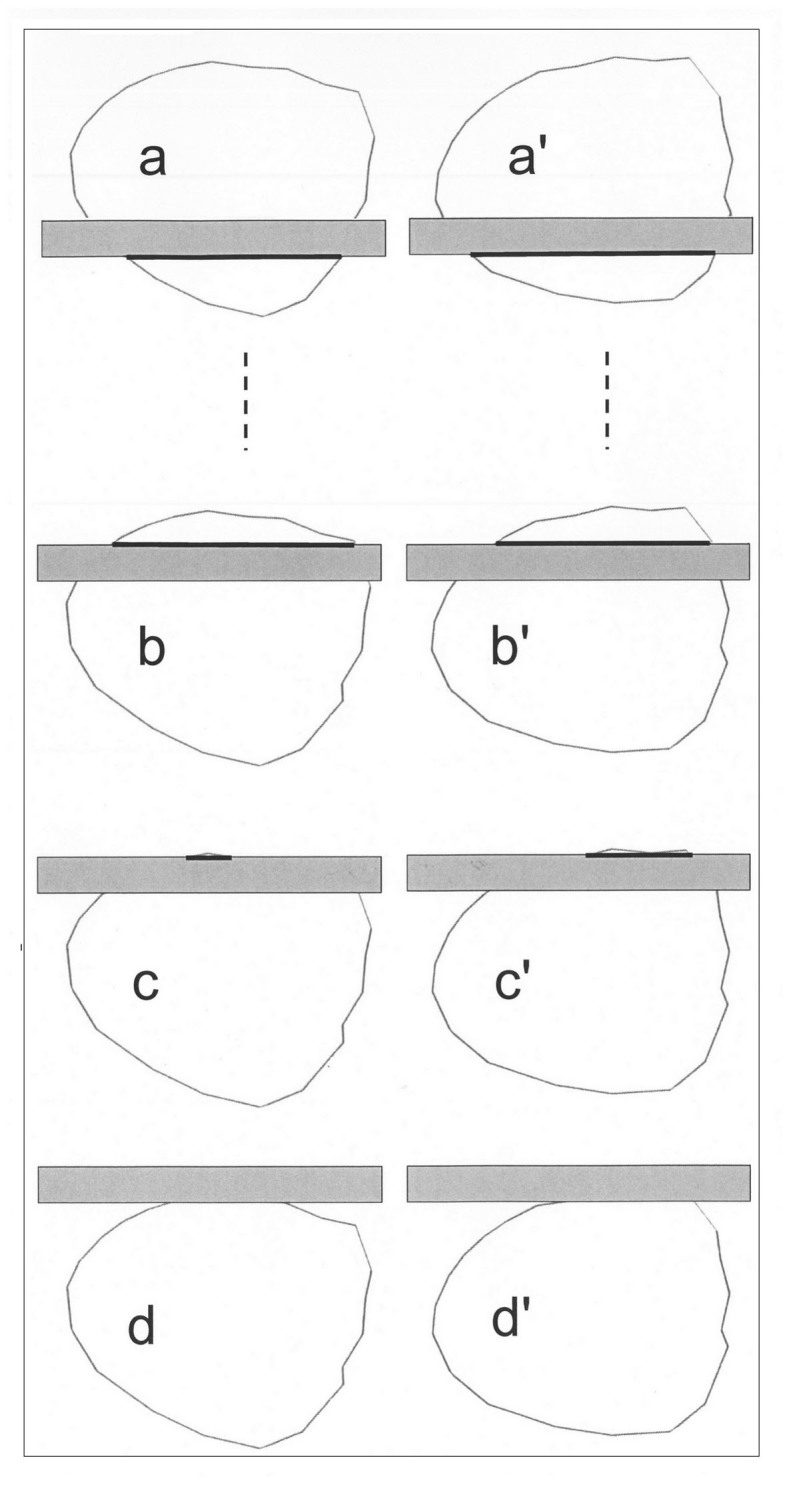
Example of eight surfaces among 100 with the form 1 of identical area to a circle with diameter of 0.470 μm. The surfaces were at distances that vary by 0.06 μm from the slice, which is 0.07 μm thick. Surfaces a′, b′, c′ and d′ were turned by an angle of 18° relative to surfaces a, b, c and d. The images of surfaces a, b, a′, b′ and c′ were the smallest sectioned sides of the slice (heavy lines). Surface c did not have an image because the smallest sectioned side of the slice was shorter than the lower image detection limit *L*_S_. Surfaces d and d′ did not have an image because they were not sectioned by the two slice sides.

Therefore, in the first phase, *D_l_*_m_ is estimated as follows. *D_l_*_m_ is assumed to be near the diameter *D_λ_*_m_ of a circle, which mean image length is *λ*_m_. Therefore, an approximate value of *D_l_*_m_ is calculated from *λ*_m_, *t* and *L_S_* using Supplementary Dataset 1 on line. Then, a number *w* of surfaces (for example, one hundred surfaces) with the form of the pore cross-sections and with the area of the circle of diameter *D_λ_*_m_ (i.e., π *D_λ_*_m_^2^/4) are drawn at different positions relative to perpendicular slices, whose thickness is *t*. The surfaces are placed at *w*^0.5^ (for example, ten) different distances, which vary by a slightly greater value than 1/(*w*^0.5^ times *D_λ_*_m_) from the slice, and the surfaces are turned by *w*^0.5^ (for example, ten) different angles, which vary by 180/*w*^0.5^ degrees (Supplementary Figs. S1, S2 and S3 on line). The image lengths, which are labelled *Λ*_0_, of the *w* surfaces are measured as shown in Fig. 4, and the mean (*Λ*_0_)_m_ of the image lengths that are longer than *L*_S_ is calculated. Note that the coefficient of variation $cv_{\Lambda_{0}}$ of these lengths can be calculated for further reference.

Assuming that the mean image length of a circle is approximately proportional to the diameter when it slightly varies from *D_λ_*_m_ to *D_l_*_m_, *D_l_*_m_ is estimated to be 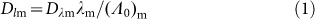
Eventually, more precise approximations of *D_l_*_m_ can be successively made using surfaces with the area of the previously estimated diameter *D_l_*_m_ (i.e., π *D_l_*_m_^2^/4) until (*Λ*_0_)_m_ becomes notably close to *λ*_m_.

Then, the mean image length *L*_m_ of the circle of diameter *D_l_*_m_ is calculated from *D_l_*_m_, *t* and *L_S_* using Supplementary Dataset 1 on line. Then, the mean length *l*_m_ of the images of the circles with identical areas as the pore cross-sections is estimated: by definition, it is equal to *L*_m_.

The coefficient of variation *cv_l_* of these images is calculated as follows (see Supplementary Note on line): 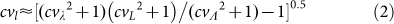
where *cv_L_* is the coefficient of variation of the image length of the circle with diameter *D_l_*_m_; *cv_L_* is calculated from *D_l_*_m_, *t* and *L*_S_ by Supplementary Dataset 1 on line. The coefficient *cv_Λ_* is the coefficient of variation of the image length *Λ* of the surface with identical area as the circle area with the estimated diameter *D_l_*_m_. In principle, it would be estimated as previously with a number of surfaces with identical area as the circle of diameter *D_lm_*. However, when *D_lm_* is close to *D_λ_*_m_, this coefficient of variation is notably close to $cv_{\Lambda_{0}}$ if *t* and *L_S_* are not too high. Therefore, *cv_Λ_* can be merely approximated using $cv_{\Lambda_{0}}$.

In the second phase, the parameters of the equivalent-diameter distribution are estimated as follows (see Supplementary Note on line): 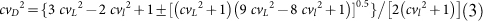
and *D*_m_ is: 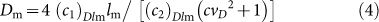
where (*c*_1_)*_Dl_*_m_ and (*c*_2_)*_Dl_*_m_ are calculated with respect to *D_l_*_m_ using equations (18) and (19) in Supplementary Note on line. Indications have been provided^10^ about selecting the sign ± in equation (3). Finally, the estimated number of pores per unit plate area, *n*_A_, is (see Supplementary Note on line): 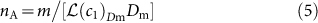
where 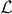 is the total slice length and (*c*_1_)*_D_*_m_, which is different from (*c*_1_)*_Dl_*_m_, is calculated with respect to *D*_m_ using equation (18) in Supplementary Note on line.

To verify that the sign ± of equation (3) is correctly selected, the distribution of the pore image length, which is calculated from the normal equivalent-diameter distribution defined by *D*_m_ and *cv_D_*, is compared to the distribution of the measured pore image lengths. For this calculation, the frequencies of the images of 100 circles with the normal diameter distribution (*D*_m_, *cv_D_*), which are obtained from 100 slices equally spaced by one hundredth of the largest diameter, are calculated. Then, the limits of each class are multiplied by the ratio *λ*_m_/*l*_m_. However, this verification may be impossible when different forms are distinguished (see below).

When q forms of pore cross-sections are distinguished, the previously described measurements and calculations are successively applied to each form. The forms are assumed present in all size classes. The calculated means of *D*_m_ and *n*_A_ are equal to the means of each form weighted by their proportions (respectively: 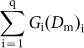 labelled 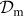 and 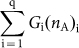 where *G*_i_ is the proportion of the form i, and i varies from 1 to q), and the coefficient of variation of the equivalent-diameter is calculated as follows (see Supplementary Note on line): 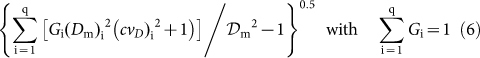
To verify that the distribution of the pore image lengths which is calculated from the estimated normal equivalent-diameter distribution, which is defined by the previous means of *D*_m_ and *cv_D_*, is comparable to the distribution of the measured pore image lengths, the procedure previously indicated to verify the selection of the root of equation (3), is applied using these means of *D*_m_ and *cv_D_*. However, the limits of each class are calculated with the ratio *λ*_m_/*l*_m_, where *l*_m_ is equal to the sum of the estimated values of *l*_m_ for each form, multiplied by the percentage of each form and the number of images of each population (calculated from equation (5)) and divided by the total number of images.

#### Estimation of the equivalent-diameter of the plates where the pores are observed

The plate image lengths are compared to image lengths of transparent circles in an opaque matrix, which are obtained from slices perpendicular to the circle plane and of thickness *t*.

With a notably small number of sieve plate images, the plate equivalent-diameter distribution is unlikely assessable and the pore cross-section form cannot be considered. Therefore, the plate images are assumed to be taken from perfectly cylindrical plates with identical diameter. This diameter, which is labelled *P*, is calculated using the mean and the coefficient of variation of the image length based on the relations in the circle (Supplementary Note on line) using Supplementary Dataset 1 on line.

With a greater number of sieve plate images, the value *L_S_* and the plate equivalent-diameter distribution can be estimated similarly to the previous case of pores from the plate image lengths.

#### Estimation of the relation between the pore equivalent-diameter and the minimal distance from the pore centre to the plate edge

When pore images are obtained from slices near the plate centre, the proportion of images obtained from the pores near the plate centre is higher than the proportion of these pores in the entire plate. Therefore, if, for example, the pores near the centre are greater, the mean pore size should be overestimated.

**Figure 5:**
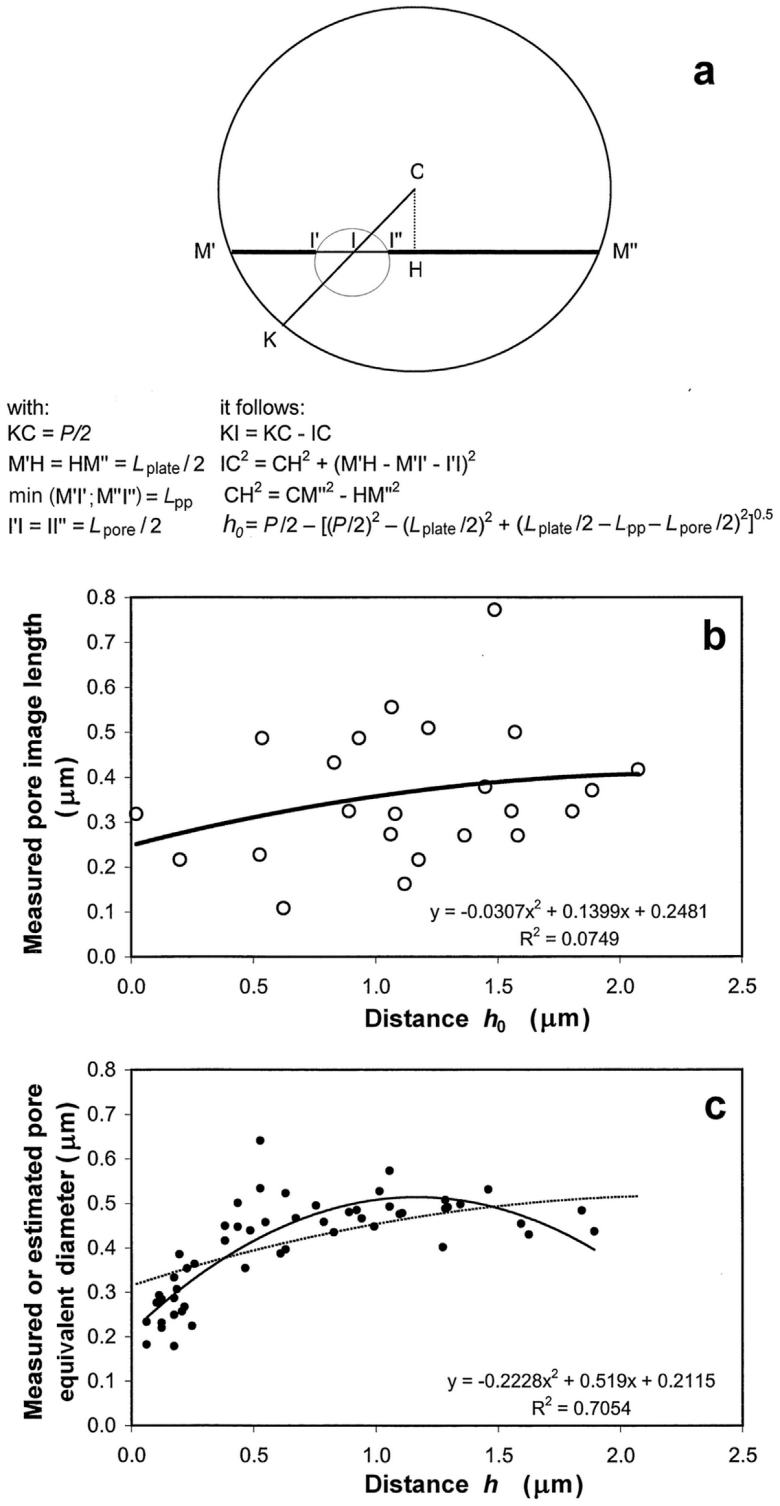
Equivalent-diameters and image lengths of the pores with respect to the distances between pores and plate edge. (a) Calculation outlines of the minimal distance *h*_0_ between the middle of the pore image and the plate edge using the plate equivalent-diameter *P*, the image lengths of the pore (*L*_pore_) and the plate (*L*_plate_) and the distances between the pore image ends and the plate image ends (*L*_pp_); (b) The measured pore image length in four longitudinal views with respect to the estimated minimal distance *h*_0_ between the pore image middle and the plate edge (**○** and regression line); (c) The measured equivalent-diameter of the pore in the transverse view in Fig. 18 from Fisher^8^ (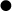 and regression line) or the estimated equivalent-diameter of the pore from equation (9) in four longitudinal views assuming three pore forms in identical proportion – hypothesis H1 – (dotted line) with respect to the measured or estimated minimal distance between the pore cross-section centre *h* and the plate edge.

The possible variation of the pore equivalent-diameter with the minimal distance *h* from the pore centre to the plate edge is assessed as follows. First, the minimal distance *h*_0_ from the pore image centre to the plate edge is calculated according to Fig. 5a using the value of *P*, the image lengths of the pores and plate, and the distance between their ends.

Let the statistic relation between the pore image length *λ* and *h*_0_ be 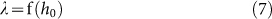
and the part of the variance of *λ* that is explained by the regression (7) be *R*^2^.

Because the expected mean ratio of the pore equivalent-diameter to the image lengths is *D*_m_/*λ*_m_, whereas the expected mean of the differences (*h*_0_ − *h*) is zero, the pore equivalent-diameter is expected vary with *h* according to the equation: 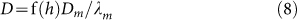


#### Proposed geometric reconstruction model to estimate the pore equivalent-diameter distribution over the entire plate

If the previous values of *cv_D_* and *D*_m_ are estimated from pore images that are obtained by slices rather near the plate centre, and if *D* tends to vary with *h*, the following geometric reconstruction model of a plate view is used to re-estimate *cv_D_* and *D*_m_ for the entire plate.

**Figure 6:**
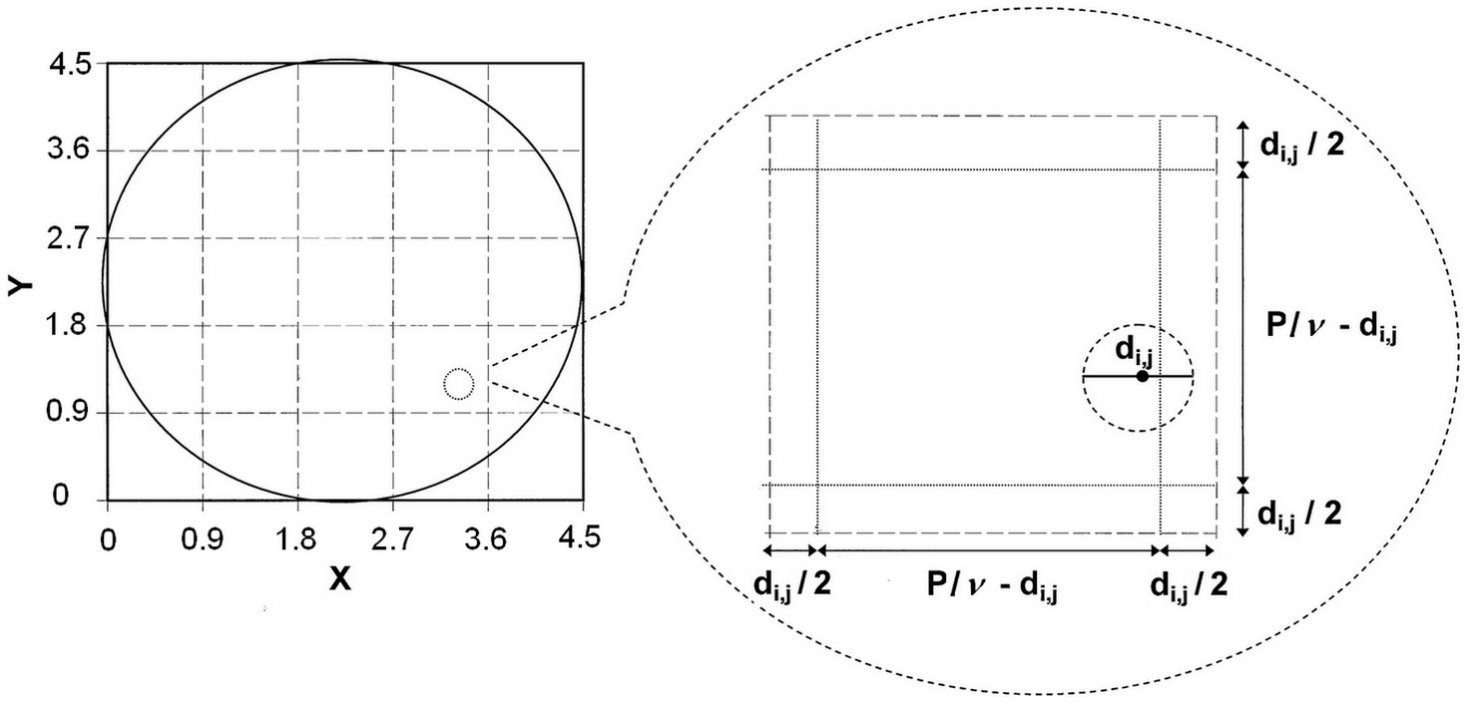
Geometric model of a plate with its pores. The circle of diameter *P* = 4.5 μm (heavy line) represents a plate in the plane (*x*, y). In the square with side *P* (heavy line), there are *ν* (here, *ν* = 5) small squares with side *P*/*ν* = 0.9 μm (dotted lines). A circle of diameter *d_i,j_*, which represents a pore cross-section (dotted line), is shown in the small square between abscissa 2.7 and 3.6 and ordinates 0.9 and 1.8 μm. This zoomed square shows the circle of diameter *d_i,j_* inside a smaller square with side *P*/*ν* − *d_i,j_*. The distance between the circle centre and the left side is *d_i,j_*/2 + *a* (*P*/*ν* − *d_i,j_*), and the distance between the circle centre and the bottom side is *d_i,j_*/2 + *b* (*P*/*ν* − *d_i,j_*), where *a* and *b* are random values between 0 and 1.

On a plane (*x*, *y*), the coordinates of the centres of the circles with diameter *d*_i,j_ (see below) which represent pore cross-sections within a circle of diameter *P* that represents a plate cross-section, are calculated as follows. In the square with centre (*P*/2, *P*/2) and side *P* (Fig. 6), *ν*^2^ squares are delimited. Each square has side *P*/*ν* and two sides that are parallel to the *y* axis, with the abscissa: 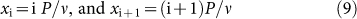
where i is an integer between 0 and *ν* − 1. The other two sides of the squares are parallel to the *x* axis, with the ordinates: 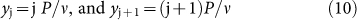
where j is an integer between 0 and *ν* − 1. In each square, a circle of diameter *d*_i,j_ is placed. This diameter *d*_i,j_ is equal to the sum of the value provided by equation (8) and a random value taken from the normal distribution with zero for mean and (1 − *R*^2^) × (*D*_m_ *cv_D_*)^2^ for variance, where (1 − *R*^2^) is the part of the variance of *λ* that is not explained by *h*_0_. The coordinates of the circle centre are: 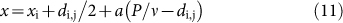

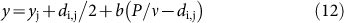
where *a* and *b* are random numbers within 0–1. However, if *d*_i,j_ is greater than *P*/*ν*, then *d*_i,j_ is equal to *P*/*ν*. If the distance calculated by Pythagoras' theorem between the centre of a circle with diameter *d*_i,j_ and the centre of the circle with diameter *P* is greater than *P*/2 − *d*_i,j_/2, the circle with diameter *d*_i,j_ is not included in the plate. The number of reconstructed circles is counted and the mean and the coefficient of variation of their diameters are calculated.

#### Estimation of the distribution of the pore equivalent-diameter over the entire plate using the proposed geometric reconstruction model

Assume that the pore cross-section centres are uniformly dispersed over the plate. Then, the estimated number of pores *n* in the entire plate is equal to *n*_A_ multiplied by the plate area, which is π *P*^2^/4: 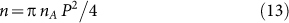
The previous proposed geometric reconstruction model is applied with some values of *ν* close to *n*^0.5^ (so that *ν*^2^ varies around *n*). A number (for example, 1000) of replications of plate reconstruction are made for each value of *ν*. The values of *D*_m_, *cv_D_* and *n*_A_ are estimated by linear inter/extrapolations of their averages, which are obtained in the reconstruction, based on *n* and the numbers of reconstructed circles.

### Validation of the proposed method

The method was tested with three datasets: one from Fisher^8^ with true longitudinal views and one transverse view from the same tissue and two from Mullendore et al.^5^ with transverse views, from which I simulated longitudinal views.

#### Dataset from Fisher^8^ and measurements

Based on a set of electron microscopic photographs of soybean leaf petiole phloem, Fisher^8^ showed that the callose formations, which may occur in the sieve plate pores, could result from artefacts because of sample preparation. This set included seven longitudinal views with an entire sieve plate section, one transverse view (Fisher's Fig. 18) with an entire sieve plate with its pores and another transverse view with a sieve tube, which is at the level of an incomplete plate with only a few visible pores (Fisher's Fig. 20). I selected four of the seven longitudinal microphotographs (Fisher's Figs. 11, 12, 21 and 24) because in the other three longitudinal views, the limits of some pores were not clearly visible or the tubes showed signs of immaturity (Fisher's Figs. 15, 22 and 23).

The image lengths of the four plates and 23 pores in the four longitudinal microphotographs by Fisher^8^ were measured, according to Fig. 1, including the callose, i.e., the white region, because it likely resulted from an artefact as reasoned by Fisher. The mean and the coefficient of variation of these lengths were calculated considering the magnification of each microphotograph. The distances of one end of the pore images to one edge of the plate images were measured.

In Fisher's Fig. 18, the contours of the cross-sections of the plate and its pores were drawn (Fig. 2a). The plate contour was delimited to the beginning of the dark cylindrical region surrounding the central part. The areas *s* of the plate or pore cross-sections were measured considering the magnification of each microphotograph. Their equivalent-diameters were calculated to be (4 *s*/π)^0.5^. The minimal distances of the centres of the circles circumscribed to the plate or pore cross-sections were measured.

In Fisher's Fig. 20, the area of the tube/plate section was measured, and the plate equivalent-diameter was calculated.

All data and calculations are provided in Supplementary Dataset 2 on line.

#### Datasets from Mullendore et al.^5^ and measurements

Mullendore et al.^5^ published two cross-sectional views of phloem sieve plates of Cucurbita and Phaseolus (Figs. 3c–d from the authors), which were obtained using electron microscopy scanning. The outlines of the plate and pore cross-sections were drawn. Based on the indicated scale, the areas of the plate or pore cross-sections were measured, their equivalent-diameters were calculated and the minimal distances of the centres of the circles circumscribed to the pores to the plate edge were measured as for the dataset from Fisher^8^.

To simulate longitudinal views of these two sieve plates on an Excel Sheet, I superimposed these images with a grid of parallel strips, with 12 μm spacing for Cucurbita or 3 μm spacing for Phaseolus, to represent the intersections with slices of thickness *t* = 0.07 μm. Then, whenever both sides of a strip section passed through a pore cross-section, the coordinates of the ends of the shortest side (Fig. 4) which was considered as the image length were noted. Similarly, the plate image length was obtained. The process was repeated for five other orientations of the grid, each of which was turned by 30 degrees with respect to the preceding position. The mean *λ*_m_ and the coefficient of variations *cv_λ_* of the image lengths of the plate and pores were calculated.

The pore cross-sections were assumed to have identical forms as in the plate from Fisher^8^. Therefore, the method was applied with three previous pore forms 1, 2 and 3.

#### Estimates obtained by applying the method to the dataset from Fisher^8^ and comparison with the true values

The estimates were firstly obtained without reconstructing the plate. Then, the equivalent-diameter of the plate and the relation between the equivalent-diameter and *h* were also estimated. The estimates were then obtained using the reconstruction model and compared to the observations.

#### Pore equivalent-diameter distribution and number of pores estimated without reconstructing the plate

**Figure 7:**
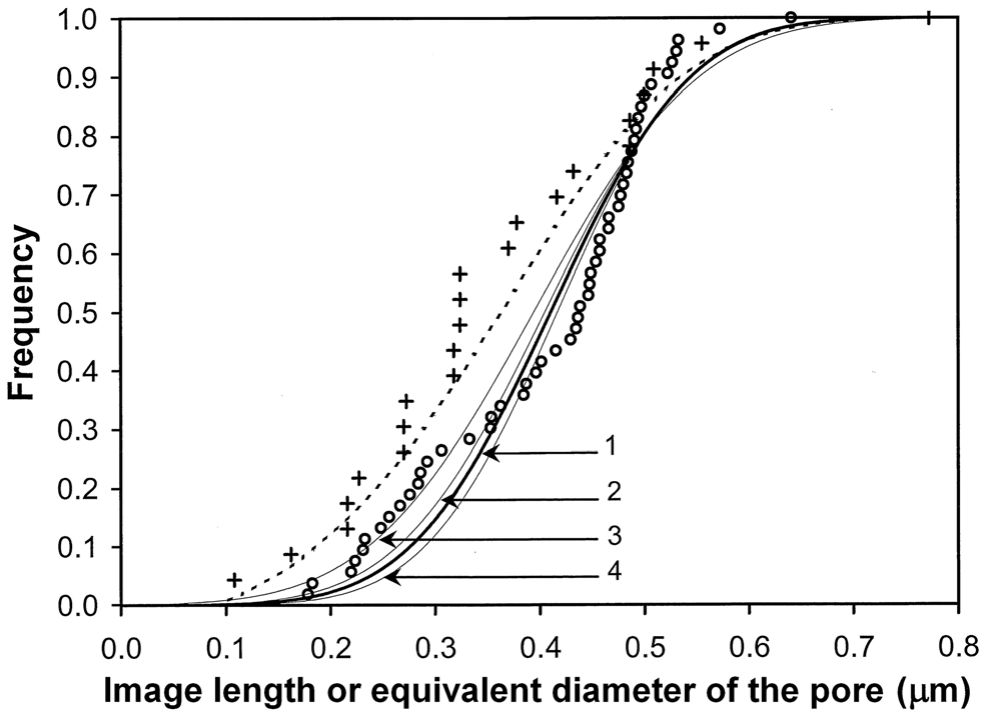
Distributions of the image lengths and the equivalent-diameters of the pores, which are measured or estimated based on the views from Fisher^8^ and in the reconstructed plates. Longitudinal views: Measured lengths of 23 pore images in four longitudinal views from Fisher^8^ (**_+_**). The distribution of the pore image lengths (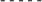) is estimated from three normal equivalent-diameter distributions (the values of *D_m_* and *cv_D_* are provided in Table 2), which are estimated for three pore cross-section forms in Fig. 2a. The normal pore equivalent-diameter distribution (heavy line 1 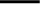; *D*_m_ = 0.411 μm and *cv_D_* = 0.256) is estimated by reconstructing the entire plate. Transversal view: Observed equivalent-diameters of 53 pores (**○**) in Fig. 18 from Fisher^8^ and the corresponding normal distribution (line 2 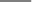; *D*_m_ = 0.405 μm and *cv_D_* = 0.272). The normal equivalent-diameter distributions of the pores are estimated using the proposed method without reconstructing the plate (line 3 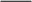; *D*_m_ = 0.394 μm and *cv_D_* = 0.314) or by reconstruction using the measured values *D*_m_ = 0.405 μm and *cv_D_* = 0.272 and the regression of the measured equivalent-diameter of the pores with the measured distance from the pore cross-section centre to the plate edge (line 4 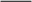; *D*_m_ = 0.418 μm; *cv_D_* = 0.232).

Based on 23 pore image lengths (Fig. 7 and Supplementary Table S1 on line), *L_S_*, *λ*_m_ and *cv_λ_* were equal to 0.093 μm, 0.359 μm and 0.413, respectively.

Based on *L*_S_, *λ*_m_ and *t* = 0.07 μm, *D_λ_*_m_ was estimated to be 0.470 μm. The pore cross-sections were successively approximated by one of the three forms observed in the transverse view (Fig. 2a) and labelled 1 (rather rounded), 2 (rather oblong) or 3 (with some concavities), which were selected to be representative of this view. One hundred surfaces with identical area to the circle of diameter *D_λ_*_m_ and shaped like one of the forms 1, 2 and 3 were drawn at 100 different positions relative to perpendicular slices, which are 0.07 μm thick (Supplementary Figs S1, S2, and S3 on line). These surfaces were placed at ten different distances, varied by 0.06 μm (which is slightly longer than one-tenth of *D_λ_*_m_) from the slice, and turned by ten different angles that varied by 18 degrees. The values of (*Λ*_0_)_m_, *D_l_*_m_, *l*_m_, *cv_Λ_* and *cv_l_* are provided in Table 2 for the three forms.

The estimates of *cv_D_*, *D*_m_ and *n*_A_ varied according to the pore cross-section form (Table 2).

Assuming that the forms 1, 2 and 3 have equal proportions (hypothesis H1), the estimated mean values of *D*_m_, *cv_D_* and *n*_A_ were 0.458 μm, 0.259 and 3.20 μm^−2^, respectively. Assuming that these proportions are 42, 30 and 28% (hypothesis H2), for further reference, these values were 0.453 μm, 0.265 and 3.24 μm^−2^. Under both H1 and H2, the estimated image length distributions were consistent with the distribution of the 23 pore image lengths, although the estimated images were slightly longer (Fig. 7).

*Estimated equivalent-diameter of the plate.* Four views were assumed to be taken from i) plates with identical size ii) at short distances from the plate cross-section centres because Fisher^8^ likely selected views that showed a relatively large number of pores, which also contributed to identifying the sieve tubes. The image lengths of the four plates in these four views were compared to the image lengths of transparent circles with identical diameter *P* in an opaque matrix, which were obtained from perpendicular slices of thickness *t* = 0.07 μm. From the mean and the coefficient of variation of these observed image lengths (Supplementary Table S1 on line), the values of *L*_S_ and *P* were calculated to be 3.589 and 5.312 μm, respectively.

*Estimated relation between the equivalent-pore diameter and the minimal pore distance to the plate edge (*h*).* The pore image length *λ* tended to increase with *h*_0_ according to the regression in Fig. 5b. The low significance level was assumed to be largely because of the random distances of the slices from the pore cross-section centres, whereas the number of pores was small. Based on this regression and equation (8), the pore equivalent-diameter (μm) was estimated to vary as shown in Fig. 5c with respect to the minimal distance *h* between the pore cross-section centre and the plate edge (μm): 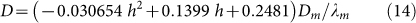
Although the regression in Fig. 5b explained only a notably small part (*R*^2^ = 0.075) of the pore image length variations, equation (14) suggested possible large variations of *D* (between 0.313 μm for pores notably near the edge and 0.509 μm for pore cross-sections near the plate centre under H2).

*Pore equivalent-diameter distribution and number of pores over the entire plate which were estimated using the proposed geometric reconstruction model.* Because the number of pores in the entire plate that was estimated using equation (13) was 71.0 or 71.8 under H1 or H2, respectively, the proposed geometric reconstruction model was applied with *ν* equal to 8, 9 and 10.

One thousand replications of the plate reconstruction were made for each value of *ν* under H1 and H2. Fig. 2b shows a replication with *ν* = 10 under H2. Under H2, the estimates of *D*_m_, *cv_D_* and *n*_A_ which were extrapolated from the means that were obtained for *ν* = 9 and *ν* = 10 (Supplementary Table S2 on line), were 0.411 μm, 0.256 and 3.24 μm^−2^. Fig. 7 shows the normal distribution of the pore equivalent-diameter based on these values of *D*_m_ and *cv_D_*. Under H1, these numbers were only slightly different (0.415 μm, 0.251 and 3.20 μm^−2^). All previous results were only slightly changed when 10,000 replications were made.

*Comparisons between the estimates obtained from the longitudinal views and the observations in the entire transverse plate view from Fisher*^8^. There were 53 pores in the sieve plate in the main transverse view published by Fisher^8^ (his Fig. 18) as previously reported and drawn in Fig. 2a. The plate equivalent-diameter was 4.647 μm. The pore cross-sections were almost uniformly distributed over the plate (Fig. 2a). The pore equivalent-diameter distribution was roughly normal (Fig. 7), and the equivalent-pore diameter tended to increase with the minimal distance from the pore cross-section centre to the plate edge according to the regression in Fig. 5c. The values of *D*_m_, *cv_D_* and *n*_A_ obtained from 53 pores were 0.405 μm, 0.272 and 3.13 μm^−2^, respectively.

The plate was reconstructed using the proposed geometric model with *P* = 4.647 μm and different values of *ν*: 8, 9 and 10. Each *d_i,j_* was equal to the sum of the value calculated by the regression in Fig. 5c and a random value from the reduced normal distribution with variance (1 − *R*^2^) × (*D*_m_ *cv_D_*)^2^, where *R*^2^ is provided in Fig. 5c, and *D_m_* and *cv_D_* are 0.405 μm and 0.272, respectively. One thousand replications of the plate reconstruction were made for each value of *ν*. Fig. 2c shows a replication with *ν* = 9. Because the number of pores over the entire plate is 53, the estimates of *D*_m_, *cv_D_* and *n*_A_, which were extrapolated from the means obtained for *ν* = 8 and 9 (Supplementary Table S2 on line), were 0.418 μm, 0.232 and 3.13 μm^−2^, respectively. Fig. 7 shows the normal distribution based on these values of *D*_m_ and *cv_D_*.

In the partial and almost transverse view of a sieve tube from Fisher^8^ (his Fig. 20), the tube equivalent-diameter was estimated to be approximately 5.4 μm.

#### Estimates obtained by applying the method to the dataset from Mullendore et al.^5^ and comparison with the true values

The values of *P*, *D*_m_, *cv_D_* and *n*_A_ that were estimated from the measured lengths *λ* of the pore and plate images obtained by the simulated slices in the two sieve plates of Cucurbita or Phaseolus phloem were notably near the true values (Table 3). The pore equivalent-diameter (μm) that was calculated from the measured pore cross-section area tended to increase with its measured minimal distance *h* (μm) to the plate edge according to the regressions *D* = 1.49 *h* + 1.09 and *D* = 0.82 *h* + 2.03 in Cucurbita and Phaseolus, although the *R*^2^ were low (0.27 and 0.04), respectively.

Because the grid was superimposed on the entire of the plate cross-section, the geometric reconstruction model was not applied.

## Discussion

The estimated plate equivalent-diameter (5.312 μm) in the four longitudinal views from Fisher^8^ was in the range (4.647–5.4 μm) of two plates that were observed entirely or partly in transverse views. This estimate was perhaps imprecise because based on only four plate images, but the consequences of this imprecision were likely limited to the number of plate pores and the diameters of the pores in the periphery.

Without reconstructing the plate, the estimates of *D*_m_, *cv_D_* and *n*_A_ obtained from the longitudinal views were 0.453 μm, 0.265 and 3.24 pores per μm^2^, respectively, under hypothesis H2. Under H1, they were only slightly different. It was verified that the pore image length distribution that was predicted from the estimates of *cv_D_* and *D*_m_ for the three pore forms by assuming normal equivalent-diameter distributions was largely consistent with the data. The observed deviation could be partly due to the equivalent-diameter distributions which were likely only approximately normal, like that observed in the transverse view (Fig. 7).

The reconstruction model was strongly validated by its application to the transverse view: i) the pore centres were uniformly dispersed over the plate (Fig. 2a); ii) the pore equivalent-diameter tended to increase with the pore distance from the plate edge; iii) the estimates of *D*_m_, *cv_D_* and *n*_A_ obtained by reconstruction were close to their true values (0.418 *vs* 0.405 μm, 0.232 *vs* 0.272 and 3.13 *vs* 3.13 pores per μm^2^, respectively).

When the reconstruction model was applied to the four longitudinal views, *D*_m_ was smaller (0.411 *vs* 0.456 μm under H2) than the value which was estimated without reconstruction, which was consistent with the higher proportion of smaller pores in the plate periphery.

Finally, the proposed method provided notably close estimates to the observed values in the transverse view (0.411 *vs* 0.405 μm for *D*_m_, 0.256 *vs* 0.272 for *cv_D_* and 3.24 *vs* 3.13 pores per μm^2^ for *n*_A_), although the number of pore images was small (23). The small difference in diameter was consistent with a study^10^ with bonbons using the Monte Carlo method with 100 simulations, where the slices were made at random distances from the bonbon centres: the deviation of the estimated bonbon diameter from its true value was lower than 5% in 95 cases of approximately 30 bonbon images. The estimates under H1 were only slightly different from those under H2.

Evidently, it is not certain that the pores were exactly comparable in the longitudinal views and the transverse view. However, the comparison heavily validates the proposed method because these views were taken from the same tissue (one to three adjacent vascular bundles of petiole). It is difficult to observe exactly identical plates in both longitudinal and transverse views.

This application to the dataset from Fisher^8^ shows the method feasibility in real observation conditions of the phloem.

The proposed method assumes that the pore cross-section form is known. The examples from the datasets from Mullendore et al.^5^ indicate that the identified forms in the plate from Fisher^8^ are sufficiently common, so the method can provide useful approximate values of the size and number of pores for many species. Incomplete cross-sectional views can provide indications about the pore form.

The reconstruction model assumes that the pores are randomly distributed over the entire plate. This assumption was acceptable in the plate of the figure from Fisher^8^. In the plates of the figures from Mullendore et al.^5^, the pore density was higher in the periphery of the plates. Therefore, the total number of pores in the plate can be underestimated by the model. Thus, it is important to observe longitudinal views with short plate images.

In conclusion, the proposed method appears able to provide even with only a few dozen images, valuable estimates of the sizes and numbers of pores from longitudinal views when the pore form is approximately known. It is expected to be useful for many studies on phloem sieve tubes and modelling sap flow, particularly on the phloem hydraulic conductivity, which likely varies with the number and the diameter of the pores according to a previously proposed equation^7^, when the pores have identical size. The method is also expected to be applicable for other tissues and materials with approximately cylindrical structures when longitudinal slices can be obtained.

## Supplementary Material

Supplementary Information Supplementary_Information

Supplementary Information Supplementary_Dataset_1

Supplementary Information Supplementary_Dataset_2

## Figures and Tables

**Table 1:** Notation of the main variables of the image length

	One circle (or several circles with identical size)			One pore cross-section (or several cross-sections with identical size)			Several circles or pore cross-sections with different sizes	
	General	Circle of diameter		General	Pore cross-section area		Circles	Pore cross-sections
		*D_l_*_m_	*D_λ_*_m_		π *D_λ_*_m_^2^/4	π *D_l_*_m_^2^/4		
Length of one image	*L*			*Λ*	*Λ*_0_		*l*	*λ*
Mean image length	*L*_m_	*l*_m_	*λ*_m_	*Λ*_m_	(*Λ*_0_)_m_		*l*_m_	*λ*_m_
cv* of the image lengths	*cv_L_*			*cv_Λ_*	$cv_{\Lambda_{0}}$	*cv_Λ_*	*cv_l_*	*cv_λ_*

cv*: coefficient of variation.

**Table 2:** Application of the proposed method to the longitudinal views from Fisher^8^

Unit of length: μm	Form	Diameter	Mean image length	cv*	n_A_* (μm^−2^)
Pores population: mean (*λ_m_*) and cv* (*cv_λ_*) of the measured image lengths			0.359	0.413	
Circle that gives the mean measured image length *λ_m_*: diameter (*D_λm_*) and *λ_m_*		0.470	0.359		
Shape with identical area as the circle of diameter *D_λm_*: mean (*Λ*_0_)_m_ and cv* ($cv_{\Lambda_{0}}$) of the measured image lengths *Λ*_0_	1		0.362	0.306	
2		0.347	0.310		
3		0.329	0.372		
Circle with identical area as the shape from which *λ_m_* would be obtained: diameter (*D_lm_*) equal to *D_λm_* *λ_m_*/(*Λ*_0_)_m_, mean (*L_m_*) and cv* (*cv_L_*) of the image length	1	0.466	0.356	0.266	
2	0.487	0.372	0.268		
3	0.513	0.392	0.270		
Shape with identical area as the circle of diameter *D_lm_*: cv* (*cv_Λ_*) of the image length, which is possibly approximated by $cv_{\Lambda_{0}}$	1			0.306	
2			0.310		
3			0.372		
Population of circles with identical areas as the pores:					
- estimated mean (*l_m_* = *L_m_*) and cv* (*cv_l_*) of the image lengths	1		0.356	0.382	
2		0.372	0.380		
3		0.392	0.322		
- estimated mean (*D_m_*) and cv* (*cv_D_*) of the equivalent-diameters and number of pores per unit area.	1	0.427		0.304	3.47
2	0.448		0.297	3.27	
3	0.498		0.176	2.88	

*cv = coefficient of variation; *n_A_* is the number of pores per unit plate area.

**Table 3:** Measured images (M) of the sieve plates and the pores of Cucurbita and Phaseolus phloem in Mullendore et al. datasets^5^ and of Soybean in Fisher datasets^8^ and the comparison between the estimated values (E) of the number of pores and the equivalent-diameter distribution, and their true values (T), which are observed in the same plate or in a neighbouring plate (the case of the Soybean dataset)

	Cucurbita			Phaseolus			Soybean		
Unit of length: μm	M	E	T	M	E	T	M	E	T
Plate									
Number of images *m*	26			33			4		
Mean length *l*_λ_	44.26			14.24			4.776		
cv*of the length *cv*_λ_	0.269			0.252			0.104		
Lower detection limit *L*_S_		7.967			3.817			3.589	
Equivalent-diameter *P*		55.81	55.14		17.79	17.63		5.312	4.647
Pores									
Number of images *m*	154			198			23		
Mean length *l*_λ_	5.264			1.244			0.359		
cv* of the length *cv*_λ_	0.420			0.389			0.413		
Shortest image length *l*_min_	0.621			0.028				0.108	
Longest image length *l*_max_	10.38			2.368				0.773	
Lower detection limit *L*_S_		0.589			0.023			0.093	
cv*of the eq-diameter *cv*_D_		0.286	0.232		0.233	0.288		0.256	0.272
Mean eq-diameter *D*_m_		6.17	6.18		1.54	1.74		0.411	0.405
Number of pores *n*		54	50		70	62		72	53
Number of pores/μm^2^ *n*_A_		0.022	0.021		0.282	0.250		3.24	3.13

*: cv = coefficient of variation; eq-diameter = equivalent-diameter.
