# Supplementary material for: Estimating the number and size of phloem sieve plate pores using longitudinal views and geometric reconstruction
Source: Sci Rep. 2014 May 12;4:4929. doi: 10.1038/srep04929 (PMC5381363; doi:10.1038/srep04929)
Supplement: Supplementary Information — Supplementary_Information [file srep04929-s1.pdf]

Estimating the number and size of phloem sieve plate pores using longitudinal views and geometric reconstruction

Philippe Bussières

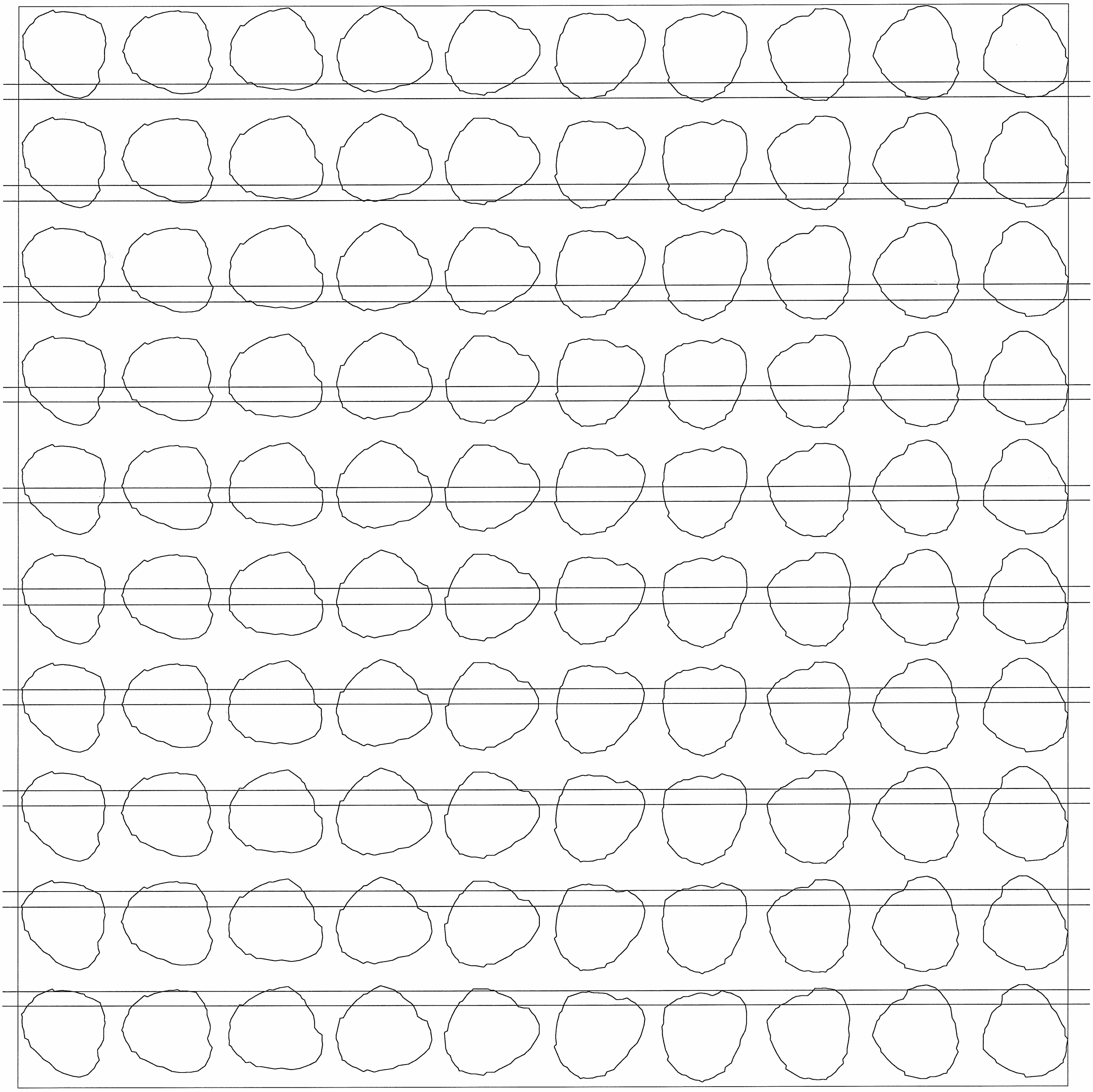

Supplementary Fig. S1. Set of one hundred surfaces with form 1, which is similar to pore 1 in Fig. 2a, with identical areas, located at ten different distances from slices that are 0.07 μm hick and turned by ten different angles.

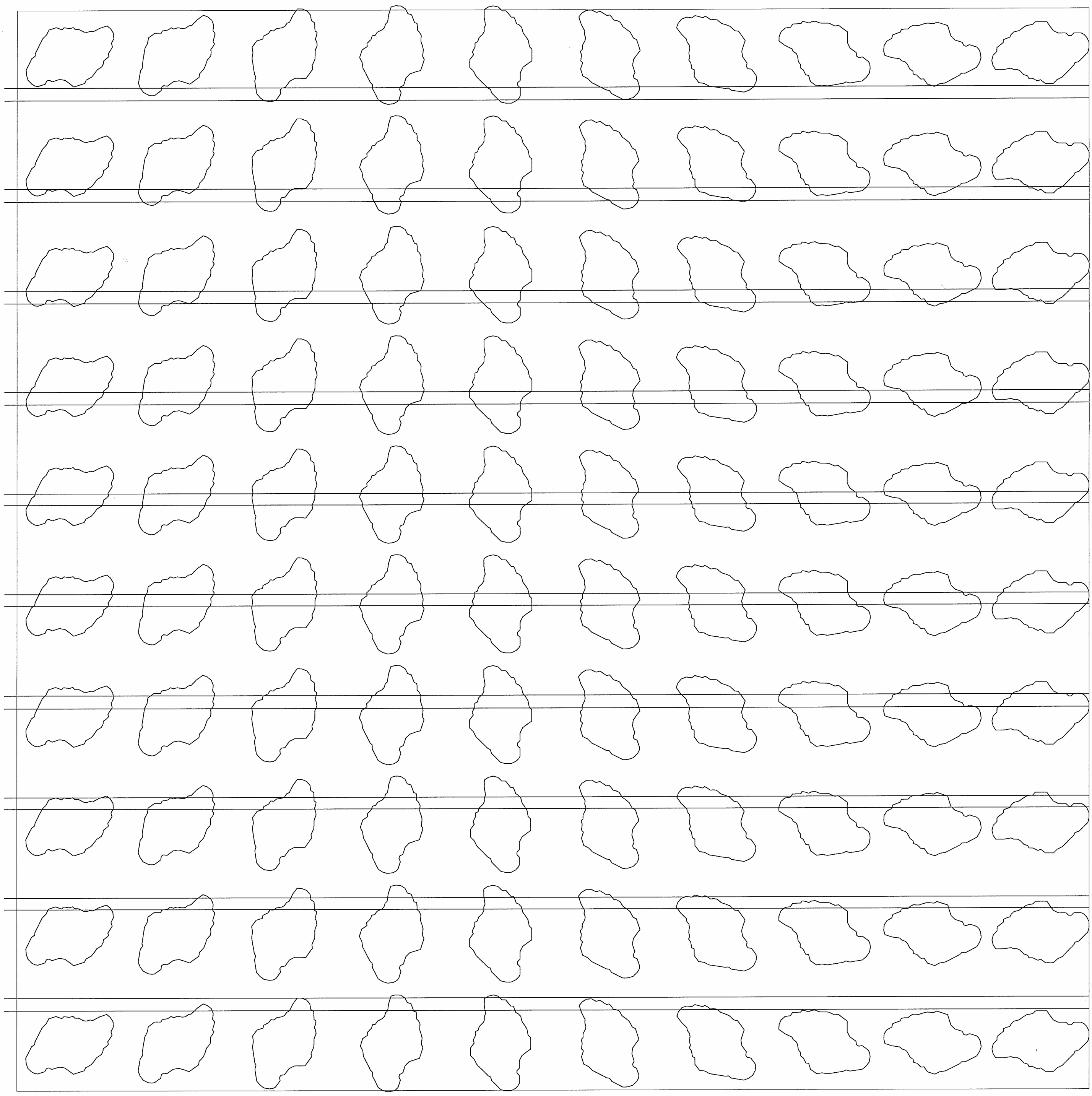

Supplementary Fig. S2. Set of one hundred surfaces with form 2, which are similar to pore 2 in Fig. 2a, with identical area, located at ten different distances from slices that are 0.07 μm thick and turned by ten different angles.

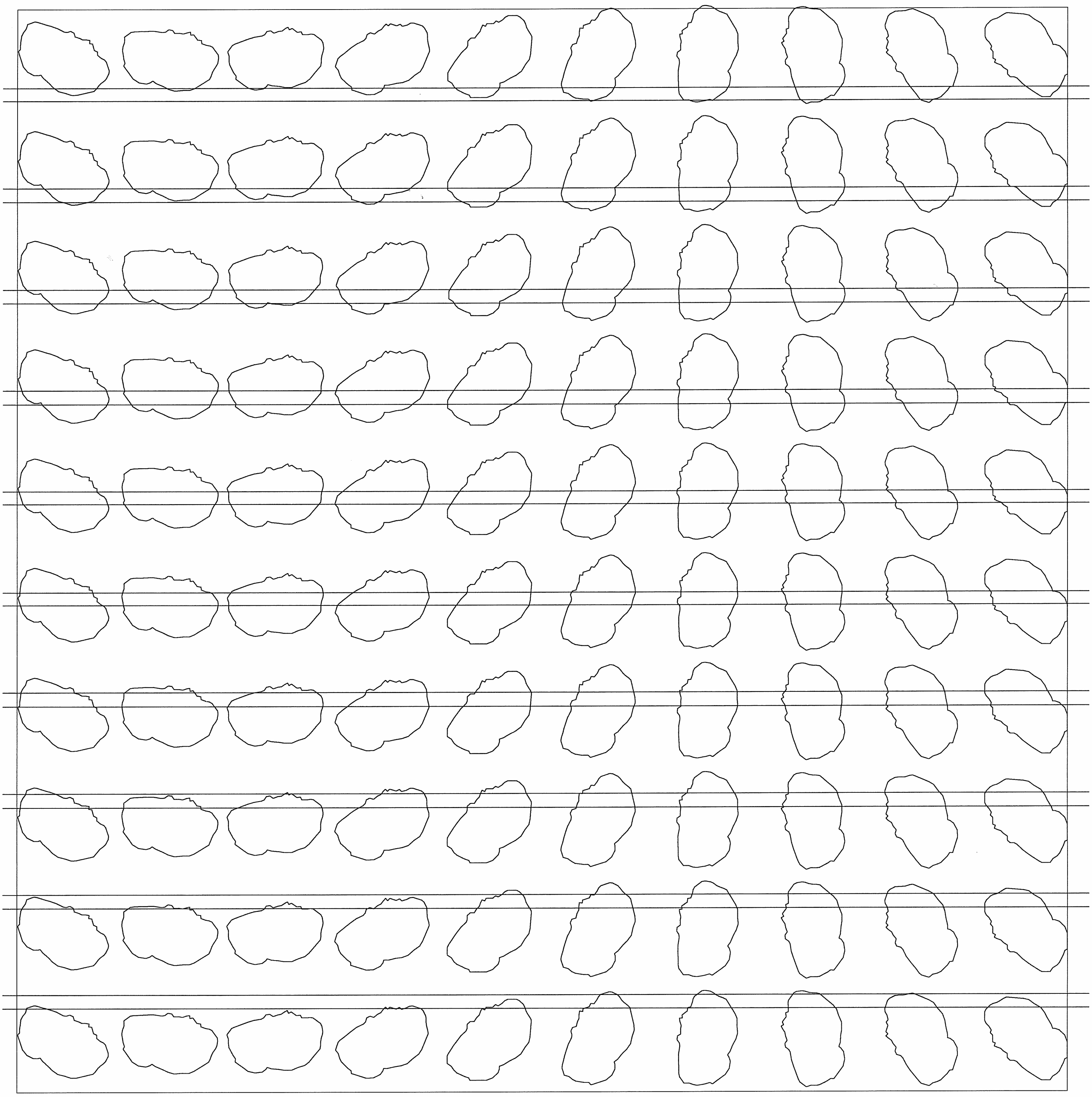

Supplementary Fig. S3. Set of one hundred surfaces with form 3, which are similar to pore 3 in Fig. 2a, with identical area, located at ten different distances from slices that are 0.07 μm thick and turned by ten different angles.

Supplementary Table S1. Values measured on the longitudinal views from Fisher[8].

	N° of the figures from Fisher[8]					
	11	12	21	24	Total or mean	cv [(1)]
Plate image length (μm)	5.000	5.185	4.054	4.865	4.776	0.104
Number of pores along the plate image	6	5	4	8	23	
Mean pore image length (μm)	0.402	0.435	0.405	0.257	0.359[(2)]	0.413[(3)]

[(1)] cv = coefficient of variation; [(2)] Mean equal to the sum of the pore image lengths divided by the number of pores (23); [(3)] cv obtained with the 23 pores lengths.

Supplementary Table S2. Pore equivalent-diameter and number of pores in the reconstructed plates from the longitudinal views or the transverse view from Fisher[8], assuming three pore forms in identical proportion (hypothesis H2).

	n		
	8	9	10
**From longitudinal views from Fisher[8]**			
Number of pores	46.3	56.20	69.5
Pore equivalent-diameter:			
mean (μm)	0.423	0.420	0.412
coefficient of variation	0.294	0.283	0.260
Number of pores per unit surface area of plate (μm^{-2})	2.09	2.53	3.13
**From the transverse view from Fisher[8]**			
Number of pores	46.4	57.4	71.1
Pore equivalent-diameter:			
mean (μm)	0.422	0.416	0.403
coefficient of variation	0.233	0.231	0.192
Number of pores per unit surface area of plate (μm^{-2})	2.73	3.38	4.19

Note

Relations among D, t, L_S, L_m and cv_L for one circle or circles with identical diameter

Based on the angles β and γ that are indicated in Figs. 3f-g, which are:

$$\gamma = \text{acos}\ (t\ /\ D) \tag{15}$$

$$\beta = \text{asin}\ (L_S\ /\ D) \tag{16}$$

it is easy to find the mean length of all images that are obtained with all possible slice positions, which is given by the circle area between the central position of the slice (Fig. 3f) and its position in Fig. 3g divided by the distance between these two positions, D (cos β – cos γ):

$$L_m = D\ c_2\ /\ (4\ c_1) \tag{17}$$

where:

$$c_1 = \cos\beta - \cos\gamma \tag{18}$$

$$c_2 = 2\gamma - 2\beta - \sin 2\gamma + \sin 2\beta, \tag{19}$$

and the coefficient of variation of these lengths is:

$$cv_L = [16\ c_1\ c_3\ /\ (3\ c_2^2) - 1]^{0.5} \tag{20}$$

where:

$$c_3 = (3 - \cos^2\beta)\cos\beta - (3 - \cos^2\gamma)\cos\gamma \tag{21}$$

With these relations, D can be obtained from t, L_S and L_m by successive approximations with decreasing values of D for a sufficiently high number of times L_m until the calculated L_m becomes lower than its true value. When L_S is unknown but t, L_m and cv_L are known, the values of D and L_S can be estimated by successive approximations with increasing values of the assumed D, for each of which, with increasing values of L_S until the calculated L_m and cv_L become lower and higher, respectively, than their true values.

Justification of equations (3), (4) and (5)

Consider a population of n circles on a plane with possibly different diameters. Let g be the function by which the fraction $g(D)$ of the population of circles with diameters between D and D + dD is related to D. This function g, which can be evaluated if the diameter distribution is known, is eliminated in the following cases. Evidently:

$$\int_{D=0}^{\infty} g(D)\, \mathrm{d}D = 1 \tag{22}$$

Consider a set of parallel slices that are spaced by a small dx and perpendicular to the plane. Assume that the circles are positioned so that their obtained images from the slices do not overlap. Denote the number of slices per unit length of the circle diameter by q, the number M of images of a circle with diameter D is equal to $q\, c_1\, D$, and the total number of images m of n circles is:

$$m = \int_{D=0}^{\infty} q\, c_1\, D\, n\, g(D)\, \mathrm{d}D \tag{23}$$

Denote the first and second moments of the image length of a circle with diameter D by L_{m} and $(L^2)_{\mathrm{m}}$. Then, the first moment l_{m} and the second moment $(l^2)_{\mathrm{m}}$ of the length of the m images are:

$$l_{\mathrm{m}} = \frac{1}{m} \int_{D=0}^{\infty} L_{\mathrm{m}}\, M\, n\, g(D)\, \mathrm{d}D \tag{24}$$

$$(l^2)_{\mathrm{m}} = \frac{1}{m} \int_{D=0}^{\infty} (L^2)_{\mathrm{m}}\, M\, n\, g(D)\, \mathrm{d}D \tag{25}$$

where M, L_{m}, and $(L^2)_{\mathrm{m}}$ vary with D. From equations (17) and (20), equations (22) and (23) give:

$$l_{\mathrm{m}} = \frac{\mathrm{A}}{\mathrm{B}} \quad \text{with A} = \int_{D=0}^{\infty} c_2\, D^2\, g(D)\, \mathrm{d}D \quad \text{and B} = 4 \int_{D=0}^{\infty} c_1\, D\, g(D)\, \mathrm{d}D \tag{26}$$

$$(l^2)_{\mathrm{m}} = \frac{\mathrm{A}}{\mathrm{B}} \quad \text{with A} = \int_{D=0}^{\infty} c_3\, D^3\, g(D)\, \mathrm{d}D \quad \text{and B} = 3 \int_{D=0}^{\infty} c_1\, D\, g(D)\, \mathrm{d}D \tag{27}$$

The squared coefficient of variation (cv_l^2) of the image length is obtained from equations (24) and (25) as:

$$cv_l^2 = \frac{(l^2)_{\mathrm{m}}}{(l_{\mathrm{m}})^2} - 1 \tag{28}$$

The variables c_1, c_2, and c_3 were found[10] to vary only slightly when the ratios D / t or D / L_{S} are high. Thus, in this case, if t and L_{S} are given, c_1, c_2, and c_3 vary only slightly relative to D and *a fortiori* to D^2 or D^3. Therefore, in equations (26) and (27), they can be factored. They can also be factored if D varies only slightly and if D / t, D / L_{S}, or both decrease. In

particular, their mean values are approximately equal to the expected value if all circles have identical diameter, which is equal to that of a circle that generates images of mean length l_m. Let D_{lm} be this diameter, the values β and γ are acos (t / D_{lm}) and asin (L_S / D_{lm}), respectively, according to equations (15) and (16), and the values of c_1, c_2, and c_3 are given by equations (18), (19) and (21). Let D_m, $(D^2)_m$, and $(D^3)_m$ be the first, second, and third moments of the diameter distribution of the circles, respectively. equations (26)-(28) provide the following approximations:

$$l_m \approx (c_2 / c_1) [(D^2)_m / D_m] / 4 \quad (29)$$

$$(l^2)_m \approx (c_3 / c_1) [(D^3)_m / D_m] / 3 \quad (30)$$

$$cv_l^2 \approx (16\, c_1\, c_3 / c_2^2) [D_m\, (D^3)_m / (D^2)_m^2] / 3 - 1 \quad (31)$$

Because the ratio 16 c_1 c_3 / (3 c_2^2) is obtained from equation (20), equation (31) becomes:

$$cv_l^2 + 1 \approx (cv_L^2 + 1)\, D_m\, (D^3)_m / (D^2)_m^2 \quad (32)$$

where cv_L is the coefficient of variation of the image length of the circle with diameter D_{lm}.

Moreover, if the diameter distribution is symmetrical, then the third central moment is nil, and it follows that:

$$(D^3)_m = 3\, D_m\, (D^2)_m - 2\, D_m^3 \quad (33)$$

Then, equation (32) gives the following approximate quadratic in (cv_D^2 + 1):

$$(cv_l^2 + 1)\,(cv_D^2 + 1)^2 - 3\,(cv_L^{\ 2} + 1)\,(cv_D^2 + 1) + 2\,(cv_L^{\ 2} + 1) \approx 0 \quad (34)$$

whose roots give equation (3). From equation (29), equation (4) becomes $(D^2)_m = (cv_D^2 + 1)$ $(D_m)^2$. Equation (23) and the previously indicated considerations[10] lead to equation (5).

Justification of equation (2)

Equation (34) can also be written as:

$$(cv_l^2 + 1)\,(cv_D^2 + 1)^2 \approx (3\, cv_D^2 + 1)\,(cv_L^2 + 1) \quad (35)$$

It is hypothesised by analogy that the following approximate equation holds if the objects are only slightly different from circles:

$$(cv_\lambda^2 + 1)\,(cv_D^2 + 1)^2 \approx (3\, cv_D^2 + 1)\,(cv_\Lambda^2 + 1) \quad (36)$$

where cv_λ and cv_Λ are the coefficient of variation of the image length of all objects and that of the image length of an object with identical surface area as the D_{lm}-diameter circle, respectively.

Equations (35) and (36) lead to equation (2).

Justification of equation (6)

Consider q statistical series of the same variable. In each series i ($1 \leq i \leq q$), use n_i to denote the number of values of the variable, m_i is their mean value, SC_i is the sum of the squares of the values, s_i is their variance, and cv_i is their coefficient of variation. Use with the same notations but without the index i for the same parameters of the population of q populations. There are the following relations:

$$cv^2 = s^2 / m^2 \quad (37)$$

$$s^2 = \sum_{i=1}^{q} SC_i / n - m^2 \quad (38)$$

$$SC_i = n_i (s_i^2 + m_i^2) \quad \text{as:} \quad s_i^2 = SC_i / n_i - m_i^2 \quad (39)$$

$$s_i^2 = m_i^2 cv_i^2 \quad (40)$$

$$cv^2 = \sum_{i=1}^{q} [n_i m_i^2 (cv_i^2 + 1) / n] / m^2 - 1 \quad (41)$$

$$cv^2 = \sum_{i=1}^{q} [G_i m_i^2 (cv_i^2 + 1)] / m^2 - 1 \qquad \text{with } G_i = n_i / n \quad (42)$$
